# The Physiology of Enteric Glia

**DOI:** 10.1146/annurev-physiol-022724-105016

**Published:** 2025-02-03

**Authors:** Jacques Gonzales, Brian D. Gulbransen

**Affiliations:** Department of Physiology, Michigan State University, East Lansing, Michigan, USA

**Keywords:** enteric nervous system, autonomic, peripheral glia, gastrointestinal, gut, intercellular communication

## Abstract

Enteric glia are the partners of neurons in the enteric nervous system throughout the gastrointestinal tract. Roles fulfilled by enteric glia are diverse and contribute to maintaining intestinal homeostasis through interactions with neurons, immune cells, and the intestinal epithelium. Glial influences optimize physiological gut processes such as intestinal motility and epithelial barrier integrity through actions that regulate the microenvironment of the enteric nervous system, the activity of enteric neurons, intestinal epithelial functions, and immune response. Changes to glial phenotype in disease switch glial functions and contribute to intestinal inflammation, dysmotility, pain, neuroplasticity, and tumorigenesis. This review summarizes current concepts regarding the physiological roles of enteric glial cells and their potential contributions to gut disease. The discussion is focused on recent evidence that suggests important glial contributions to gastrointestinal health and pathophysiology.

## 1. INTRODUCTION

The enteric nervous system (ENS) is an extensive network of neurons and glia in the intestinal wall that controls moment-to-moment gastrointestinal functions (1). There is a relative division of labor between the two principal interconnected ganglionated networks that make up the ENS in which the myenteric (Auerbach’s) plexus primarily regulates intestinal motility, while the submucosal (Meissner’s) plexus controls mucosal functions (2). There is also a division of labor between the two cell types housed within the ENS: enteric neurons and glia. Neurons have well-defined roles in transducing and transmitting signals that underlie complex gut functions, while the roles of their glial partners have remained less defined. What evidence has emerged indicates that enteric glia have a central role in maintaining ENS homeostasis.

The term enteric glia was introduced by Giorgio Gabella (3) in 1981 to distinguish glia in the ENS from other populations of peripheral and central glia based on their unique ultrastructural characteristics. Prior to this, enteric glia were described as early as the nineteenth century by Dogiel (4), who referred to them as satellite cells adjacent to enteric neurons. Works in the intervening time frequently noted the presence of glia in enteric ganglia but referred to these cells as Schwann cells (5), with the assumption of similarities with other peripheral glia. However, unique morphological and biochemical aspects suggested properties that were unlike those of other peripheral glia and potentially more similar to those of astrocytes (6). Enteric glia express a glial fibrillary acidic protein (GFAP) variant shared with astrocytes, express S100 calcium-binding protein B (S100B) (7), and display protoplasmic and fibrous shapes similar to white and gray matter astrocytes (8). Enteric glial activity encoded by intracellular calcium (Ca^2+^) responses (9, 10) also contributes to modulating neuronal circuits and functions in the intestine (11, 12). However, certain genetic markers of astrocytes such as Aldh1L1 do not appear to be highly expressed by enteric glia, and enteric glia exhibit gene expression profiles that are distinct from those of other types of central or peripheral glia (13–16).

The field of enteric glial biology is rapidly evolving, and new discoveries continue to expand the breadth of processes that involve glial contributions. This review summarizes many of the known functions of enteric glia with a focus on the latest research discoveries. Enteric glia are gaining notoriety for serving critical roles in various intestinal functions and pathologies; yet, many glial functions remain mysteries that, if unraveled, could hold important insight into mechanisms that control gut physiology and disease.

## 2. ORIGINS AND HETEROGENEITY OF ENTERIC GLIA

### 2.1. Developmental Origins of Enteric Glia

The ENS predominantly originates from neural crest cells in somites 1 to 7 of the vagal crest (17). These cells begin their migration at embryonic day (E)9 in mice and follow a rostrocaudal path to populate the entire intestinal tract. Cells from somites 1 and 2 colonize the esophagus and upper stomach, while cells from somites 3 to 7 extend from the stomach to the colon. Sacral crest–derived cells may also contribute to the ENS in the distal intestine, including the glial lineage (18), but the extent to which the sacral crest contributes to ENS formation has been challenged by recent findings (19). By E15.5, precursor cells have reached their final destination in the gut and initiate a centripetal migration to form what will become the myenteric plexus and, subsequently, the submucosal plexus (20).

Once in position, neural crest cells choose whether to commit to either neural or glial fates. Initially, enteric neural precursors express the transcription factors SRY-box transcription factor 10 (Sox10) and Ret. Cells committing to a neural fate continue to express Ret and decrease Sox10, while those destined to become enteric glia maintain Sox10 and reduce Ret expression (21). In mice, enteric glia undergo various maturation stages during this period of cellular differentiation marked by the appearance of glial markers such as fatty acid–binding protein 7 (B-FABP) (E11.5), proteolipid protein 1 (PLP1) (E12.5), S100B (E14.5), and GFAP (E16) (22–25). These maturation processes are not exclusive to mice and occur in a similar manner in other animal species. Similar to the ENS as a whole, enteric glia undergo significant postnatal maturation that involves increases in GFAP and S100B expression (26). Some subsets of extraganglionic glia such as those in the lamina propria and mucosal villi do not appear until postnatal day 10 in mice (27). This final migration coincides with the colonization and establishment of the gut microbiota, which promote the development of mucosal glia (27, 28).

In adult mice, mucosal glia continue to be replenished from cells of the myenteric plexus in a process influenced by the intestinal microbiota. Disruptions to the gut microbiome lead to changes in enteric glial numbers within the mucosa, but this effect is transient and normal glial composition is restored after the reestablishment of intestinal flora (27). Despite the continual replenishment of mucosal glia, the rate of gliogenesis within the myenteric plexus is low under physiological conditions. Data from bromodeoxyuridine (Brdu) and 5-ethynyl-2′-deoxyuridine (EdU) labeling demonstrate that only 1% of GFAP^+^ cells in the colonic myenteric plexus and 2.8% of S100B^+^ cells in the ileal myenteric plexus exhibit proliferation in steady-state conditions (29, 30). Mechanisms of gliogenesis are regulated in part by phosphatase and tensin homolog (Pten) signaling, and inhibiting this pathway increases the proportion of EdU^+^ glial cells in healthy mice (31). Whether cells incorporating these markers of proliferation are newly formed glia or enteric progenitors supporting turnover is undefined, and these techniques do not capture cells that transdifferentiate or migrate to the ENS from extrinsic innervation (32).

### 2.2. Enteric Glial Heterogeneity

The term enteric glia currently encompasses all glia within the gastrointestinal tract. This includes glia associated with enteric neuron cell bodies and processes in the plexus, with enteric neuron processes that extend to the mucosa and smooth muscle coats, and with extrinsic nerve processes that innervate the gut. Several criteria have been proposed to delineate subpopulations of enteric glia based on morphology, anatomical location, or marker expression (7, 8, 12). These concepts were summarized in an excellent recent review (7), so they are not reiterated here. More recent single-cell genetic sequencing data and functional imaging studies add complexity to concepts of glial heterogeneity (15, 16, 33–35). These studies suggest important differences in glial functions and interactions with surrounding cells.

**Figure 1:**
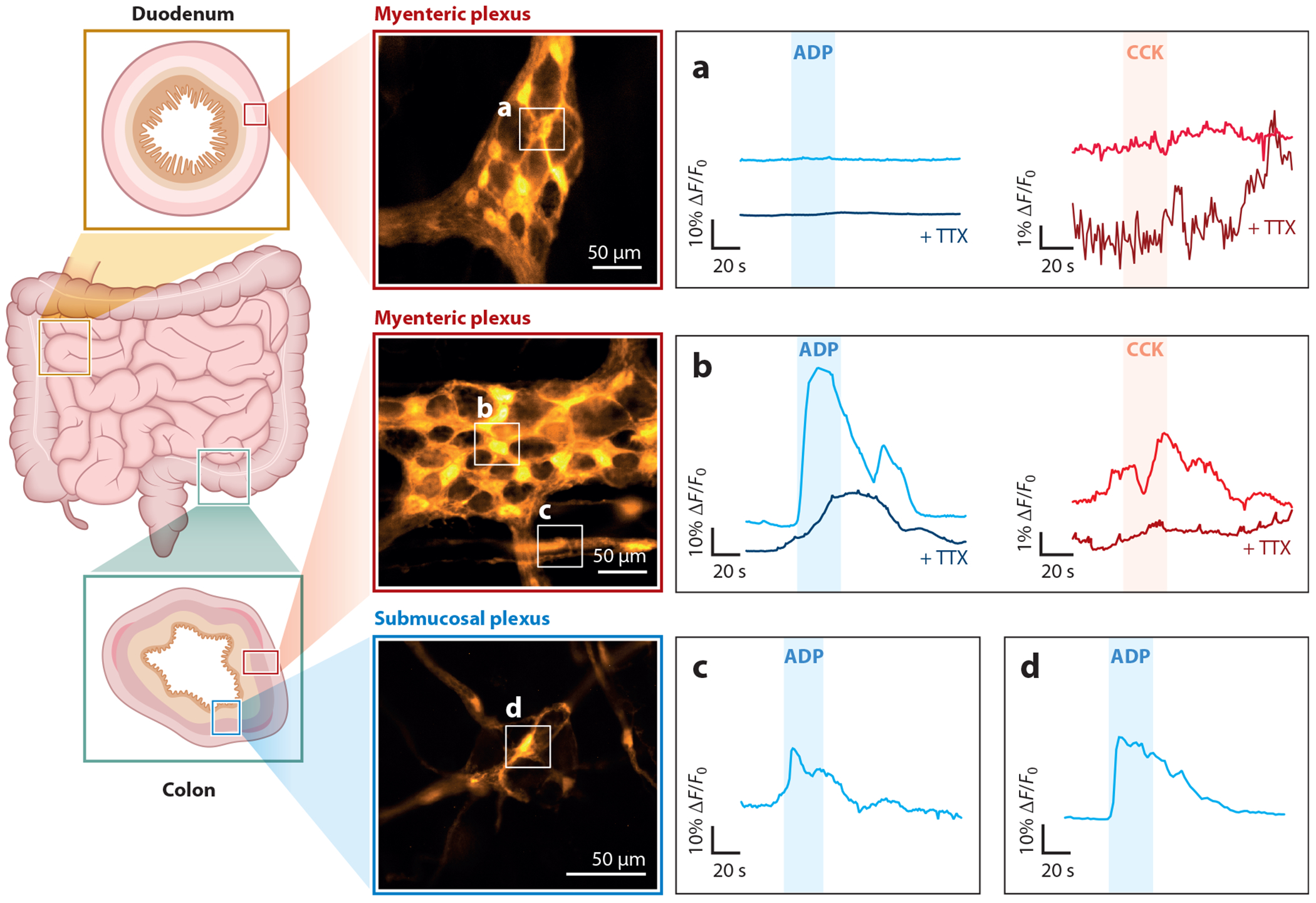
Heterogeneity in enteric glial responses within and between gastrointestinal regions. (*a–d*) The light blue curves illustrate glial responses to ADP; duodenal myenteric glia (*a*) display different dynamics when compared to glia in the colonic myenteric plexus (*b–c*) and submucosal plexus (*d*). In the colon, intraganglionic myenteric glia demonstrate a notably stronger reaction to ADP (*b*). (*a–b*) Dark blue curves demonstrate that ADP-induced glial responses are influenced by neuronal cross talk. The neuronal cross talk varies significantly across different gastrointestinal regions. The light red curves denote the glial response to CCK stimulation, while the dark red curves show the response to CCK stimulation in the presence of TTX. CCK elicits larger variability between duodenal and colonic myenteric glia. Abbreviations: ADP, adenosine diphosphate; CCK, cholecystokinin; TTX, tetrodotoxin.

Enteric glia exhibit activity encoded by intracellular Ca^2+^ responses, and mechanisms downstream of glial Ca^2+^ responses are involved in modulating neural pathways that control gut motility and secretions (9, 35–39). The neurons, neurotransmitters, and pathways responsible for triggering glial Ca^2+^ responses differ both within and between regions of the gastrointestinal tract, and glial Ca^2+^ responses display differences in their kinetics between glial cell subtypes (34, 35). For example, glial responses to the purine adenosine diphosphate (ADP) and the peptide hormone cholecystokinin (CCK) are distinct in the duodenal myenteric plexus from those in the colon myenteric plexus and involve cross talk with neurons to differing extents (34) (Figure 1a,b). Glia in the duodenal myenteric plexus exhibit variability in their responses to CCK and ADP, while glia in the myenteric plexus typically display more homogenous strong responses to ADP (34). Purine-evoked glial responses are also stronger in glia located within myenteric ganglia than extraganglionic glia (40) (Figure 1b,c). In vitro studies indicate that purinergic responses may be more pronounced in mucosal/submucosal glia compared to myenteric glia; however, this observation has yet to be confirmed in intact tissue (41). Differences in purinergic responses between myenteric glia also contribute to differential effects of glia on neural pathways within the same region of the myenteric plexus (35). The myenteric neurocircuits underlying peristalsis and other motor patterns involve overlapping ascending and descending neural pathways that promote oral contraction and aboral relaxation through fast purinergic and cholinergic synaptic signaling (42, 43). Functionally specialized subpopulations of myenteric glia are devoted to each pathway and exert contrasting effects on neural circuits depending on whether glial activity is evoked by purines or acetylcholine (35). Glial activity recruited by purinergic signaling appears to potentiate ascending circuits, while cholinergic glial recruitment exerts potential cross-inhibitory actions that function to dampen activity within descending neural networks. These observations suggest significant functional heterogeneity among the enteric glia that underlie specialized interactions with surrounding neurons with different functionally relevant outcomes.

Different classes of enteric glia also exhibit heterogeneity in their expression of canonical glial markers such as GFAP, S100B, PLP1, and Sox10 (13, 40) (Table 1). Notably, within the myenteric plexus, most intraganglionic glial cells marked by enhanced green fluorescent protein expressed under the control of the *Plp1* promoter also coexpress S100B, Sox10, and GFAP. However, extraganglionic glia typically coexpress S100B and Sox10 but less frequently GFAP (13). Whether differences observed in glial marker expression reflect stable differences between glial populations or transient fluctuations due to ongoing adaptations to environmental cues is unknown. To what extent changes involve differences in gene expression versus relevant differences in protein is also unclear. For instance, while the *Plp1* gene is widely expressed by peripheral glia and serves as a reliable marker for identifying enteric glia in the intestine, it encodes for two distinct proteins: PLP1 and DM20 (13, 44). Notably, DM20 expression predominates in nonmyelinating Schwann cells and enteric glia (44, 45). This indicates that although *Plp1* gene expression is a reliable marker to identify enteric glia, the protein expression of PLP1 itself may not label all enteric glia populations.

Genomic sequencing of enteric glia on a cell-by-cell basis provides added depth into the molecular makeup of enteric glia but produces variable results related to heterogeneity. Cell clustering analysis indicates up to seven distinct genomic subtypes of enteric glia in some studies, while only two are observed in others, which primarily reflect glial gene profiles in health and during a pathophysiological insult (15, 16, 33, 46). Some of this variability is due to differences between species, intestinal organs, and isolation, purification, and analysis techniques. The extent of stable genetic variability between subtypes of enteric glia is unclear. Glia are dynamic cells, and ongoing adaptations to maintain homeostasis may produce significant variability even within a single cell. These features of glia are further complicated by limitations of single-cell RNA sequencing that include incomplete transcriptome coverage and the loss of spatial and temporal information due to a unique transcriptomic snapshot (47). These limitations are being overcome by advances in sequencing techniques that allow sampling RNA from single cells over time (48) and assessing the spatial distribution of transcripts in tissue (47). Nonetheless, it is clear that genomic sequencing data need additional validation to understand their functional relevance and reproducibility. Similar issues regarding enteric neuron single-cell sequencing data were recently highlighted in a consensus perspective by experts in this area (49).

## 3. ENTERIC GLIA ROLES IN GASTROINTESTINAL PHYSIOLOGY

### 3.1. Neuronal Support

Enteric glia are inextricably tied to neurons in anatomy and function (50). Similar to all glia, one of the main roles of enteric glia is to optimize neuronal function (51, 52). Regrettably, few studies have taken mechanistic approaches to investigate glial support of neuronal function in the gut, but several indicate roles for glial neurotrophins. Enteric glia produce neurotrophic factors such as nerve growth factor (NGF), proepidermal growth factor (proEGF), and glial cell line–derived neurotrophic factor (GDNF) (53–55). Neuron-glia coculture models show that the presence of glia enhances synaptic connections and increases the complexity and branching of the neuronal network, which mirrors the effects observed with astrocytes. These effects appear to be driven by GDNF and purinergic signaling and are lost when GDNF is neutralized or when P2Y_1_ purinergic receptors are blocked (53).

A second way by which enteric glia maintain neuronal functions is by regulating the availability of neurotransmitters. Immunolabeling data show that enteric glia express neurotransmitter precursors such as l-arginine (56, 57) and glutamine (58, 59). This finding could indicate that glia sustain neurotransmission by shuttling these precursors to neurons, but functional evidence supporting this concept is lacking. Enteric glia do regulate the availability of neurotransmitters through their expression of uptake transporters and enzymes that degrade neuroactive compounds such as purines, peptides, and gamma-aminobutyric acid (GABA) (60–66). While the roles of peptide uptake and GABA transporters (GATs) have not been thoroughly tested, glial purine degradation does modify enteric neuron numbers and function. The extracellular purine-degrading enzyme nucleoside triphosphate diphosphohydrolase-2 (NTPDase2) is primarily expressed by glia in the digestive tract, and mice lacking NTPDase2 exhibit a loss of nitrergic myenteric neurons and defects in inhibitory junction potentials in the colon (60, 63, 65). These mice also exhibit more severe intestinal inflammation and defective barrier function following acute colitis. Therefore, glial regulation of purine availability plays essential roles in normal homeostasis and responses to disease.

Oxidative stress is a major mechanism that contributes to enteric neuropathies. In vitro and in vivo data show that enteric glia serve important antioxidant defense roles, in part by secreting glutathione (67, 68). Enteric glia produce glutathione and can adjust their glutathione synthesis through autocrine mechanisms whereby 15-deoxy-Δ-^12,14^-prostaglandin J2 (15d-PGJ2) boosts intracellular glutathione levels (69). Enteric glia also secrete *S*-nitrosoglutathione (GSNO), known for its potent antioxidant properties in central neurons, but its effectiveness in reducing oxidative stress within enteric neurons remains unconfirmed (68, 70, 71). In the ENS, glutathione synthesis appears necessary to support normal enteric neuron survival at a steady state since inhibiting this mechanism in the myenteric plexus in vitro leads to neuronal death through mechanisms that involve P2X_7_ purinergic receptors (67). However, components of the glutathione biosynthesis machinery are shared between enteric neurons and glia, and it is unclear whether specifically targeting glial contributions would have the same effect. Neurons in the brain also have limited capacity for glutathione synthesis but rely on their glial counterparts to supply key precursors (72, 73). It is likely that a similar arrangement exists between enteric neurons and glia, but the relative contributions remain unclear.

The potential for enteric glia to contribute to enteric neurogenesis has been a major area of interest, and new data suggest broad neurogenic capabilities under certain circumstances. In vitro, isolated glial cells can differentiate into multilineage colonies similar to neural stem cells (29). The cells derived from these cultures express pan-neuronal markers such as beta-tubulin III or HuC/D; express neurotransmitters such as neuronal nitric oxide synthase, vasoactive intestinal peptide (VIP), neuropeptide Y, and calbindin; and present functional synapses (29, 30, 74, 75). In vivo, genomic sequencing of mature enteric glia reveals that a distinct subpopulation of glia within the myenteric plexus still encodes genes for transcription factors of neural differentiation such as *Phox2b* and *Ascl1* (46, 75). These cells exhibit an open chromatin structure at neuronal promoters, suggesting a latent capacity to revert to a neurogenic state (75). Glia poised for neurogenesis express GFAP and are housed within myenteric ganglia, but whether extraganglionic glia share this neurogenic potential is unclear (13, 46). Despite the inherent potential of glial cells to form new neurons, regular ongoing neurogenesis from enteric glia under steady-state conditions is limited (29, 30, 46). The neurogenic potential of glia is activated following inflammation as indicated by lineage tracing of *Sox2*^+^ and *Plp1*^+^ glial cells. Neurons derived in this manner express calretinin, which would suggest an excitatory phenotype, and form functional synapses (30, 76). Given that enteric neuropathies are common in many gastrointestinal diseases, harnessing the neurogenic capabilities of glia could represent an attractive therapeutic strategy.

### 3.2. Roles for Enteric Glia in Enteric Motor Neurocircuits

Patterns of gastrointestinal motility such as colonic motor complexes, peristalsis, and segmentation are controlled by neural circuitry housed in the myenteric plexus (42, 77, 78). These circuits involve intrinsic primary afferent neurons, interneurons, and motor neurons that project in ascending excitatory pathways and descending inhibitory pathways. Myenteric glia surround neuronal cell bodies and processes within these circuits and exhibit bidirectional communication with neurons during colonic motor responses (9, 37) that function to refine neuronal circuits and optimize gut motility (35, 37).

A role for enteric glia in regulating intestinal motility was suggested by early evidence showing reduced gastric emptying and slow intestinal transit in ablation models that targeted GFAP^+^ glia (70, 79). Similarly, impairing all glial metabolism with fluoroacetate impairs intestinal transit and reduces the efficacy of neuromuscular contractions (80, 81). Unlike the differential impact of various glial subpopulations on intestinal barrier function (described later in this review), depleting any of the *Gfap*^+^, *Plp1*^+^, or *Sox10*^+^ glial populations slows gastrointestinal transit (82). This observation seems to indicate that glia play a more prominent role in gut motor neuro-circuitry than in epithelial functions. One mechanism by which glia exert control over enteric neurons is through intercellular signaling mediated by glial connexin-43 channels (38). Deleting glial *connexin-43* slows multiple aspects of gut motility and impairs both neurogenic contractions and relaxations. This overall dampening of motor responses is contrasted by effects observed when glia are stimulated by Gq-coupled chemogenetic DREADD receptors (35, 37, 39). This approach mimics endogenous muscarinic receptor activation and stimulates glial activity encoded by Ca^2+^ responses similar to those observed following neuronal stimulation. Using this approach to stimulate enteric glia leads to an increase in colonic transit and enhanced aspects of colonic motor complexes such as a 25% increase in frequency, a 55% increase in velocity, and an increase in amplitude (37, 39).

Effects of enteric glia on motor circuits involve the actions of glial subpopulations that are functionally devoted to specific neural pathways (35). Most, if not all, myenteric glia exhibit intracellular Ca^2+^ responses when neuronal activity is driven by a nonselective stimulus such as electrical field stimulation. In the colon, these glial responses are mediated by neurotransmitters such as adenosine 5′-triphosphate (ATP) and acetylcholine, which are also the primary neurotransmitters involved in fast synaptic transmission in the myenteric plexus (39, 43, 83–86). Enteric glia detect these neurotransmitters on a synapse-by-synapse basis, and overlapping ascending and descending neural circuits recruit activity in different pools of enteric glia. Many glia are responsive to stimuli that preferentially activate multipolar intrinsic primary afferent neurons, while smaller, nonoverlapping pools respond specifically to unipolar ascending and descending pathways (35). Interestingly, blocking glial activation by cholinergic mechanisms reduces circuit specificity among neurons and glia, while blocking glial activation by purinergic mechanisms reduces the efficacy of ascending pathways with opposing actions on descending circuits. Using a chemogenetic DREADD approach to mimic glial cholinergic activation constrains both ascending and descending pathways, which agrees with the loss of specificity observed when this mechanism is blocked. Overall, these results indicate that neuron-glia communication in myenteric neurocircuits is more refined than previously thought and that glia modulate neural pathways underlying motility through mechanisms that imply synaptic precision and functionally specialized subsets of cells.

**Figure 2:**
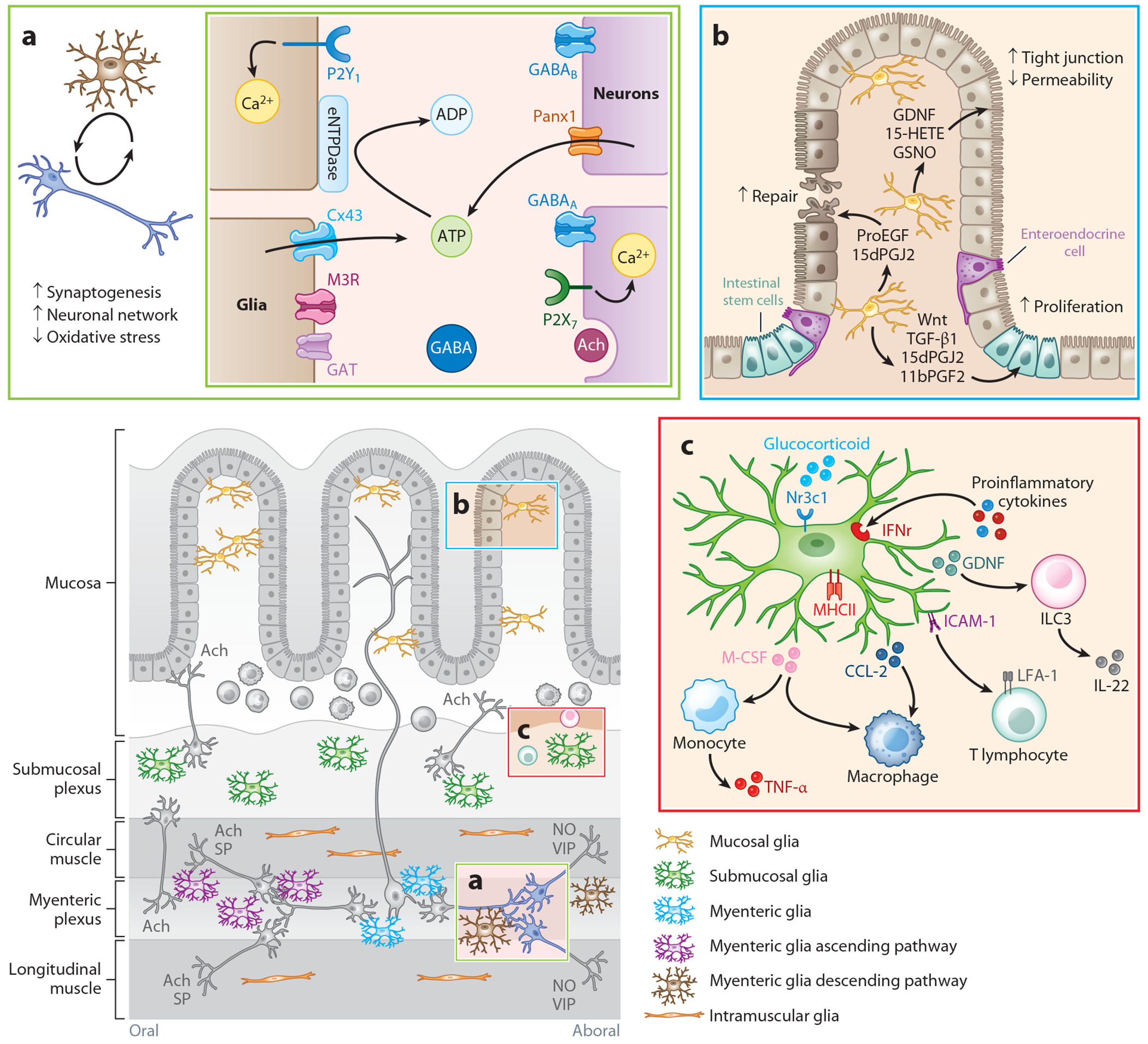
Enteric glia contribute to the maintenance of intestinal homeostasis. (*a*) Bidirectional communication between enteric neurons and glia within the myenteric plexus supports neuronal functions and regulates intestinal motility. (*b*) Enteric glia support intestinal epithelium integrity and repair through the secretion of glia-derived mediators. (*c*) Enteric glia modulate immune cell recruitment through chemokine release or direct physical interactions. Abbreviations: 11bPGF2, 11-β-prostaglandin F2; 15-HETE, 15-hydroxyeicosatetraenoic acid; 15dPGJ2, 15-deoxy-Δ-^12,14^-prostaglandin J2; Ach, acetylcholine; ADP, adenosine diphosphate; ATP, adenosine 5′-triphosphate; Ca^2+^, calcium; CCL-2, monocyte chemoattractant protein-1; CM, circular muscle; Cx43, connexin-43; eNTPDase, ectonucleoside triphosphate diphosphohydrolase 2; GABA, gamma-aminobutyric acid; GAT, GABA transporter; GDNF, glial cell line–derived neurotrophic factor; GSNO, *S*-nitrosoglutathione; ICAM-1, intercellular adhesion molecule 1; IFNr, interferon receptor; IL-22, interleukin-22; ILC3, group 3 innate lymphoid cell; LFA-1, lymphocyte function–associated antigen 1; LM, longitudinal muscle; M, mucosa; M3R, muscarinic M3 receptor; M-CSF, macrophage colony-stimulating factor; MHCII, major histocompatibility complex class II; MP, myenteric plexus; NO, nitric oxide; Nr3c1, nuclear receptor subfamily 3 group C member 1; P2X_7_, purinergic receptor P2X 7; P2Y_1_ , purinergic receptor P2Y 1; Panx1, pannexin-1; proEGF, proepidermal growth factor; SMP, submucosal plexus; SP, substance P; TGF-β1, transforming growth factor-β1; TNF-α, tumor necrosis factor-alpha; VIP, vasoactive intestinal peptide.

Glial cells play a critical role in modulating extracellular neurotransmitter levels in the myenteric plexus by regulating transmitter release, degradation, and reuptake (Figure 2a). Glial influences are particularly apparent with purinergic neurotransmission in the ENS (43). Neuronal activity stimulates glial response through cholinergic and purinergic mechanisms in which acetylcholine acts on glial muscarinic M3 receptors, or purines such as ADP act on glial P2Y receptors (10, 39). Intracellular signaling downstream of M3 and P2Y_1_ receptors couples to a rise in intracellular Ca^2+^ that subsequently causes ATP release into the extracellular space through glial connexin-43 hemichannels (10, 39, 84). This mechanism of gliotransmission is active under physiological conditions; however, potentiation of glial ATP release through connexin-43 hemichannels during disease contributes to neuroinflammation, neuronal damage, and nitrergic neuron death (87, 88). Glia contribute to constraining extracellular levels of ATP through the actions of the cell surface ectonucleotidase NTPDase2, which degrades ATP to ADP (60). Mice lacking NTPDase2 exhibit reduced nitrergic neurons, diminished neurogenic intestinal relaxation, and increased inhibitory junction potential amplitude in smooth muscles. These consequences demonstrate a role for enteric glia in modulating intestinal motility by regulating purinergic signals within the myenteric plexus.

Enteric glia can also contribute to GABA release and uptake. GABA exerts both excitatory and inhibitory effects in the ENS through the activation of ionotropic GABA_A_ or metabotropic GABA_B_ receptors, respectively (89) (Figure 2a). In the central nervous system, astrocytes express GAT to clear excess neurotransmitters from synaptic spaces (90). Immunohistochemical data suggest that enteric glia also express GAT, although their precise function remains less defined (91). One suggested mechanism is that enteric glial activation promotes GABA release through transporter reversal (64). This mechanism is similar to those observed in astrocytes where glial ATP release depolarizes the glial cell membrane and induces transporter reversal (67, 90). However, enteric glial GABA uptake, synthesis, and release mechanisms require additional characterization and validation before the roles of glia in enteric GABAergic signaling can be understood.

An emerging theme is that enteric glia could contribute to sex differences observed in gastrointestinal motility disorders. Female bias is a common clinical observation in gastrointestinal motility disorders, and data from glial ablation models and cellular imaging studies suggest a greater role of glia in motor circuits in female mice (92, 93). Genetic ablation of *Plp1*^+^ glia does not affect gastrointestinal transit time in males, but it significantly impairs transit in females (93). Likewise, the frequency of colonic motor complex in females is typically lower than in males, but glial depletion tends to equalize frequencies between sexes. It is likely that this difference is due to differential impacts of enteric glia on neuronal motor pathways. Cellular imaging studies in mice show that stimulating myenteric neurocircuits recruits similar numbers of glia in both sexes; however, responses triggered in female glia are stronger than those in males (35). This sex difference is maintained in chemogenetic models in which glial activation is mediated by Gq-coupled DREADD receptors. Stimulating glia in this model system produces greater effects on neurons in females and reduces descending motoneuron recruitment to a greater extent than in males. This finding suggests that the descending cholinergic circuitry is subject to sex-dependent modulation by enteric glia. The underlying reasons for these sexual differences remain to be fully understood but likely involve both genetic and hormonal influences. Enteric neurons and glia express estrogen receptors, including ERα, ERβ, and GPER (94), and the GPER agonist 17β-estradiol inhibits neurogenic contractions in females (94, 95). Additional work is needed to understand how similar mechanisms contribute to sexually dimorphic glial functions and their effects on intestinal motility.

### 3.3. Enteric Glia and Epithelial Functions

The intestinal epithelium is a selectively permeable barrier that provides the first line of defense for the host against the external environment. The barrier structure is established through robust cell-cell interactions that control intercellular space and regulate intestinal permeability (96). Furthermore, the epithelium is continuously renewed, shedding 10^11^ cells daily to defend against pathogens and eliminate damaged cells (97).

The intestinal mucosa is endowed with a rich population of enteric glia that are housed at crypt bases and extend projections to the villus tip (54, 98, 99). Several glia ablation models have been studied to understand the role of these mucosal glia in maintaining intestinal barrier function with varying results. The first ablation models focused on depleting *Gfap*^+^ cells using either the herpes simplex virus thymidine kinase–ganciclovir approach or an autoimmune reaction against GFAP^+^ cells (100, 101). Severe intestinal inflammation, death, epithelial disruption, and crypt hypertrophy following glial ablation all suggested essential roles for enteric glia in maintaining barrier function (99). However, methodological limitations raised questions regarding whether the observed effects were due to normal roles of glia or non-cell-autonomous responses that confound interpretations (93, 102). These questions were addressed in later studies by using a genetic approach in which diphtheria toxin subunit A (DTA) was expressed in *Plp1*^+^ cells to deplete enteric glia and limit impacts on neighboring cells (93). Despite depleting most mucosal enteric glia, this approach did not alter epithelial structure or permeability or produce crypt hyperplasia. Newer data provide a potential explanation for these apparently conflicting results. Interestingly, depleting either *Gfap*^+^ or *Plp1*^+^ glial populations using the DTA approach had little effect on intestinal barrier function (33). However, severe inflammation and death associated with a loss of barrier function were produced when both *Gfap*^+^ and *Plp1*^+^ cell populations were depleted in tandem. These observations suggest that glial subpopulations have redundant effects on the epithelium, with the loss of one population being compensated for by the other. Interestingly, ablating *Gfap*^+^ cells, but not the *Plp1*^+^ population, led to decreased expression of intestinal stem cell markers, including *Lgr5*, *Olfm4*, *Axin1*, and *Lrig*, at crypt bases and ectopic crypt progenitor proliferation. This effect suggests that *Gfap*^+^ mucosal glia play a particularly important role in maintaining stem cell renewal and intestinal epithelial homeostasis (33). The effects of *Plp1*^+^ cells on the epithelium may be more nuanced and related to aging. Mice that lack *Plp1* gene expression have normal gut permeability at 3 months but decreased permeability at 1 year of age (103). It is currently unclear whether these effects are due to changes in enteric glia or defects in myelination caused by the loss of *Plp1* expression in other glial cells. Further work is needed to understand the relevance of PLP1 in enteric glia.

In vitro coculture systems show more consistent effects of enteric glia on epithelial cell lines. For example, coculturing enteric glia with Caco-2 cells alters Caco-2 transcriptomes within 24 h to differentially regulate up to 116 genes (104). Affected genes imply glial influences on several aspects of epithelial cell function, including cell cycle, growth, proliferation, morphology, and death. This gene expression alteration results in an overall enhancement of barrier function as reflected by lower permeability to small molecules ranging from 0.4 to 4 kDa, increased transepithelial electrical resistance, and higher expression of tight junction proteins that are localized to the borders of epithelial cell membranes (70, 99, 105–110). Glia also appear to enhance epithelial repair processes by facilitating epithelial cell migration and wound repair in vitro (54, 111). These effects involve paracrine signaling mediated by glial mediators such as GDNF (109, 112), GSNO (70, 106, 113), lipid-derived metabolites [i.e., 15-hydroxyeicosatetraenoic acid (15-HETE)] (111, 114, 115), transforming growth factor-β1 (TGF-β1) (99), and proEGF1 (54) (Figure 2b). *Gfap*^+^ glia increase in vivo following injury and also participate in early epithelial regeneration by secreting Wnt ligands that enhance intestinal stem cell functions (33). The extent to which effects observed in culture extend to in vivo scenarios is not entirely clear, but several pathways have been validated in animal models or human biopsies, supporting the potential for glia to influence the intestinal epithelium (110).

Enteric glia also influence epithelial ion transport, presumably through interactions with secretomotor neurons. Early studies that impaired glial metabolism with fluoroacetate were unable to find evidence for glial contributions in secretomotor function (80). However, later work revealed a role for glia that involves effects of glia-derived nitric oxide (NO) on secretomotor neurons (116). Further, limiting glial intercellular communication by deleting glial *connexin-43* decreases ion transport up to 75% without affecting epithelial permeability (36). Interestingly, mimetic peptides targeting connexin-43 hemichannels were unable to produce the same effects, which could indicate issues with peptide access to glial channels or nonhemichannel effects mediated downstream of the connexin-43 deletion model (117). However, effects of glia on secretomotor function can also be evoked by stimulating glial cells using a targeted chemogenetic approach. Here, data show that stimulating enteric glia through Gq-coupled DREADD receptors promotes electrogenic ion transport that is mediated by interactions with secretomotor neurons and possibly direct interactions with the epithelium (36).

### 3.4. Enteric Glia and Immune Homeostasis

The gut is the largest immune organ, and interactions between immune, neural, and microbial components need constant tuning to maintain homeostasis. Enteric glia are gaining recognition for fulfilling immune signaling functions in health and disease. While most evidence is derived from pathophysiological contexts, there is a growing appreciation that ongoing glia–immune system interactions are important in normal homeostasis. Glia express machinery necessary to detect pathogens, secrete both pro- and anti-inflammatory mediators, and participate in immune cell recruitment (118–121) (Figure 2c).

One of the most basic defensive functions of enteric glia involves ENS barrier function. Enteric glial processes delineate the ganglionic borders and contribute to forming the extracellular matrix barrier that surrounds enteric ganglia (122). This barrier exhibits similarities to the blood-brain barrier and filters interactions between intraganglionic cells and the external environment (122). Resident macrophages are present at openings in the barrier and may survey the environment within enteric ganglia, similar to microglia in the brain (122–125). These macrophages play important roles in refining synaptic connections and organization of the ENS during development and maintain reciprocal interactions with enteric neurons and glia in mature animals that support ENS functions (126).

Glia contribute to influencing muscularis macrophage phenotype in adult mice by secreting TGF-β and macrophage colony-stimulating factor (M-CSF), which promote neuroprotective and proinflammatory phenotypes, respectively (99, 121, 126, 127) (Figure 2c). Glial-derived chemoattractants such as monocyte chemoattractant protein-1 (CCl-2) also act to recruit macrophages during the resolution of inflammation and guide their differentiation toward an anti-inflammatory phenotype (128). Enteric glia contribute to activating resident muscularis macrophages during active inflammation through mechanisms that involve glial connexin-43 hemichannels and production of M-CSF (129). Similar mechanisms appear to be activated by acute stress (121). Stress hormones such as catecholamines and corticosteroids interact with glial nuclear receptor subfamily 3 group C member 1 (Nr3c1) glucocorticoid receptors and promote M-CSF release and subsequent monocyte recruitment. Interfering with this pathway decreases gut inflammation in response to stress.

Enteric glia modulate innate immune responses at the level of the intestinal mucosa. Here, group 3 innate lymphoid cells (ILC3s) contribute to antimicrobial defenses, and their activity is regulated by various signals, including neuropeptides, hormones, eicosanoids, and cytokines (130). ILC3s protect against colitis through mechanisms that involve Ret signaling (131). This ILC3 mechanism appears to involve a glial component whereby GFAP^+^ cells near intestinal crypts produce GDNF after sensing environmental signals through Toll-like receptors (TLRs) (82, 131). Subsequent Ret signaling in ILC3 controls IL-22 production and protects against intestinal inflammation. These observations suggest a role for enteric glia in modulating ILC3 activity, but more work in this area is needed.

Enteric glia also contribute to modulating gut adaptative immune responses. In vitro coculture systems show that enteric glia secrete factors that suppress both CD4^+^ and CD8^+^ T lymphocyte proliferation (132). However, achieving this effect requires maintaining an unusually high lymphocyte-to-glia ratio of 1:1, which raises questions about the in vivo relevance of this effect. Enteric glia may also protect T lymphocytes against apoptosis by secreting interleukin (IL)-7 (133). Both of these observations seem to suggest a role for glia in promoting T cell maturation. Enteric glia also modulate T lymphocyte activation during acute inflammation in mice by inducing major histocompatibility complex class II (MHCII) expression (134). Glial MHCII seems less involved in presenting material phagocytosed from the extracellular environment and is instead connected with glial autophagy mechanisms. The outcome of activating T cells further strengthens contacts between lymphocytes and enteric glia mediated through intercellular adhesion molecule 1/lymphocyte function–associated antigen 1 (ICAM-1/LFA-1) interactions, which could influence the local immune environment of enteric neurons (135, 136).

## 4. ENTERIC GLIA IN PATHOPHYSIOLOGY

Given the diverse roles of enteric glia in maintaining neural, epithelial, and immune homeostasis in the gut, it should come as no surprise that glia are also active players in disease processes (12). Glial responses in these contexts can be beneficial or harmful depending on the nature and severity of the insult, disease stage, glial subtype, and other factors. Activated, reactive, and gliosis are terms used to describe different glial states in disease, and each has a different connotation. Activated refers to glial responses to external cues during physiological processes, while reactive refers to a glial response to pathophysiological insults of any severity. Gliosis refers to a dysfunctional response and/or survival of enteric glia, and gliopathy is a permanent maladaptation of glia, often associated with detrimental effects.

Pathophysiological roles of enteric glia become evident in inflammatory conditions such as inflammatory bowel disease (IBD) and irritable bowel syndrome (IBS), as well as neurodegenerative diseases, including Parkinson’s disease (PD) (137–139). In these contexts, glial function or reactivity can exacerbate local inflammation, contribute to dysfunctional barrier integrity, and alter neuronal function and immune response. Roles of enteric glia in physiopathology were covered in several recent comprehensive reviews, and readers are referred there for additional details (137–139). The following sections offer a brief overview of exemplary conditions in which enteric glia are implicated in pathophysiological processes (Table 2).

### 4.1. Enteric Glia in Inflammation

Gut inflammation triggers defensive reactions by enteric glia that are context dependent. The classic response is characterized by increased GFAP expression and the secretion of proinflammatory molecules such as IL-1β, IL-6, IL-10, interferon gamma (IFN-γ), and tumor necrosis factor-alpha (TNF-α) (140–143). However, a more thorough reactive enteric glial gene signature has been proposed based on genetic sequencing data (144). Glial responses may promote or constrain gut inflammation depending on the stage, severity, and nature of the insult. This affects the repertoire of glia-derived mediators secreted and subsequent responses in effector cells. Such changes may contribute to pathophysiology or be beneficial. As an example, IBD is marked by glial reactions reflected by increased GFAP and S100B in inflamed regions of the gut (138). In this context, reactive enteric glia modify their panel of secreted mediators such as GDNF, GSNO, 15dPGJ2, 15-HETE, and ATP, which have both beneficial effects on the gut epithelium and positive or negative effects on neurons (55, 109, 111, 114, 140).

Enteric glia are active in processes leading to enteric neuroplasticity during inflammation. Intrinsic and extrinsic primary afferent neurons release tachykinins during the acute phase of inflammation that act on neurokinin-2 receptors expressed by myenteric neurons and glia (145). Neuron responses to the tachykinin neurokinin-A promote ATP release through pannexin-1 channels and responses in the surrounding enteric glia. Subsequent responses in enteric glia increase NO production through inducible NOS, which enhances glial ATP release through connexin-43 hemichannels (146). Glia-derived ATP acts on neuronal P2X_7_ receptors and promotes neuron death and neuroinflammation (87, 145). Concurrently, changes in glial phenotype lead to the secretion of glia-derived mediators and proinflammatory molecules such as IL-1β, IL-6, and prostaglandin E2 (PGE2), which promote further neuronal plasticity (147–149). This local secretion of proinflammatory cytokines affects connexin-43 gating in glia and facilitates the release of mediators such as PGE2, which increase production during intestinal inflammation (149–151). The increased concentration of PGE2 triggers activity in VIP^+^ secretomotor neurons in the submucosa (152) and enhances the neuronal excitability of myenteric neurons (148). This enhancement of neuronal activity is associated with gastrointestinal disorders characterized by abnormal epithelial secretion and defects in propulsive motility (150).

### 4.2. Enteric Glia in Visceral Pain

Visceral pain is the most common complaint in gastroenterology and is driven by processes that sensitize afferent, nociceptive neurons during intestinal inflammation. Several pieces of evidence suggest that enteric glia contribute to nociceptor sensitization and visceral pain during acute and chronic gut inflammation in mice. First, disrupting glial metabolism with fluoroacetate attenuates visceral hypersensitivity following colitis in mice (153). This effect likely involves multiple populations of central and peripheral glia since fluoroacetate is a nonspecific metabolic poison for many glia, and the decrease in visceral hypersensitivity was associated with a decreased expression of transient receptor potential cation channel subfamily V member 1 (TRPV1) and S100B in the myenteric plexus, dorsal root ganglia, and the brain (153). However, data from more targeted approaches strengthen support for enteric glial roles in pain and identify potential mechanisms. These mechanisms include direct effects on nociceptive nerves and indirect actions through effects on immune cells. During chronic colitis in mice, enteric glia increase production of M-CSF through a mechanism that requires glial connexin-43 (127). Glial M-CSF acts on nearby macrophages to influence their phenotype, and interfering with this mechanism protects mice from developing visceral hypersensitivity during colitis. It is likely that this mechanism contributes to links between stress and inflammation in visceral pain given that psychological stress influences gut macrophage phenotype through effects on glial M-CSF secretion (121). Enteric glia also have the potential to directly influence nociceptive nerve terminal sensitivity in the gut through production of proinflammatory cytokines such as IL-1β and IL-6 during acute colitis in mice (149). These mediators promote glial connexin-43 gating and facilitate gliotransmitter release. Under these conditions, enteric glia increase production of PGE2, which sensitizes nociceptive nerves through actions on EP4 receptors expressed by TRPV1^+^ nociceptors. Blocking glial connexin-43 with mimetic peptides or deleting glial connexin-43 using targeted genetics in mice prevents sensitization of TRPV1^+^ nerve fibers in isolated tissue preparations and visceral hypersensitivity in vivo following acute inflammation. These observations suggest major roles for enteric glia in mechanisms underlying visceral hypersensitivity, but additional work in this area is needed to understand how glia contribute to visceral hypersensitivity in various contexts, whether the actions of glia on nociceptors are beneficial or harmful, and if mechanisms studied in mice are conserved in humans.

### 4.3. Enteric Glia in Dysmotility Disorders

Postoperative ileus (POI) is a prevalent gastrointestinal motility disorder characterized by reduced intestinal movement due to physical manipulation of the small intestine during surgery. Enteric glia have recently emerged as central players in gut responses to physical manipulation. Interestingly, glial responses in animal models of POI are mediated by sympathetic innervation through β-adrenergic signaling (144). Glial responses are characterized by two phases of gene expression in which an initial proinflammatory phase promotes a reactive glial phenotype that promotes IL-1β signaling and enhances intestinal inflammation by recruiting macrophages and driving IL-6 release (154, 155). Glial proinflammatory responses are enhanced by ATP, which activates glial p38-MAPK and reinforces the reactive phenotype (156). Blocking glial activation by ATP through P2X_2_ purinergic receptors reduces inflammation following surgical manipulation and the gliosis phenotype in human samples. The second phase of glial gene expression during POI is associated with the expression of genes related to proliferation and migration (144). These changes correlate with increased GFAP expression and glial proliferation.

Glial activation by endothelin is also an important mechanism contributing to POI in mice (157). Myenteric glia express the endothelin B receptor, and intestinal manipulation increases receptor expression in the small intestine. Endothelin-1, the predominant agonist of endothelin B receptors, is expressed in the varicosities of the myenteric neurons adjacent to enteric glia. Activating endothelin B receptors induces a transient relaxation of circular muscle associated with a decrease in muscle contraction and an inhibition of peristalsis. These outcomes could be explained by either inhibitory actions on excitatory cholinergic pathways or excitatory actions on inhibitory nitrergic pathways. The two possibilities are not mutually exclusive, and it is likely that glia play a part in the effects given that intestinal manipulation upregulates glial endothelin B receptors. Further studies of these mechanisms could clarify the pathophysiology of POI and identify new therapeutic targets.

Chronic intestinal pseudo-obstruction (CIPO) is another severe motility disorder in which enteric glia are emerging as important contributors. The mechanisms involve signaling by lysophosphatidic acid (LPA) through glial lysophosphatidic acid receptor 1 (LPAR1). LPA is a potent cue for both central and peripheral glia and can influence responses including cell migration, proliferation, and cellular phenotype. In microglia, LPA acts as a proinflammatory molecule through LPAR1 and induces neuroinflammation (158). *LPAR1* is the most abundant GPCR gene expressed by enteric glia, and LPAR1 protein expression is confined to enteric glia in the ENS of mice and humans. Interestingly, enteric glial LPAR1 expression is reduced in samples from humans with CIPO (159). Studies in mice suggest that the glial LPA/LPAR1 signaling pathway regulates ENS function through strong effects on glial Ca^2+^ responses and subsequent effects on neurogenic contractions. Blocking LPA signaling through LPAR1 in vivo produces major changes to ENS architecture, abnormal gastrointestinal motility, and, in some cases, a failure of gut motility that is reminiscent of CIPO in humans. These observations suggest important roles for glial LPA signaling in ENS maintenance and normal gut functions, but further work in this area is needed.

### 4.4. Enteric Glia and Intestinal Infection

Parasitic and bacterial infections are a major cause of gastrointestinal disorders and promote intestinal inflammatory responses, epithelial damage, and neuroinflammation through mechanisms that involve enteric glia (160, 161). For example, intestinal infection with *Heligmosomoides polygyrus* leads to the recruitment of IFN-γ-producing immune cells. The IFN-γ/IFN2 receptor signaling in enteric glia triggers the secretion of chemokine interferon-γ inducible protein 10 (CXCL10), which contributes to tissue repair after infection (118). Enteric glia are also involved in parasitic infections caused by *Trypanosoma cruzi* in Chagas disease, which produces gastrointestinal dysfunction and dilatation of the esophagus or colon (162, 163). In Chagas, a reduction of glial cells in the dilated megacolon is observed prior to neuronal degeneration. Conversely, glia exhibit features of a reactive phenotype in nondilated segments of colon that include increased GFAP expression, upregulation of antigen-presenting molecules, and expression of costimulatory molecules. The acquisition of this reactive phenotype could indicate a protective response against inflammation induced by the infection, but further research is needed to confirm the precise role of glia.

Enteric glia can differentiate pathogenic bacteria from probiotics through the expression of TLR (164). Pathogenic bacteria trigger TLR signaling in enteric glia which subsequently signal through the S100B/RAGE pathway to promote inflammation. In the context of *Clostridium difficile* infection, glia undergo senescence and death but also play a role in modulating intestinal inflammation through the S100B/RAGE signaling pathway (165, 166). Glial S100B is an important molecular effector in these circumstances, and S100B levels are elevated in human and animal colonic tissues infected with *C. difficile* (165). *C. difficile* toxins A and B upregulate S100B/RAGE signaling, which increases the secretion of proinflammatory cytokine IL-6 and neutrophil recruitment in the mucosa (165). The inhibition of S100B with pentamidine leads to a reduction in the intestinal damage and diarrhea severity associated with *C. difficile* infection.

Enteric glia also are susceptible to viral infections such as human immunodeficiency virus (HIV), John Cunningham virus, and SARS-CoV-2 (COVID-19) and modulate the host responses (167–170). For example, individuals infected with HIV often report severe diarrhea attributed to the viral protein HIV-1 trans-activating factor (HIV-1 Tat). HIV-1 Tat activates enteric glia, which leads to a neuroinflammatory response mediated by TLR4, S100B, and inducible nitric oxide synthase signaling (169). In addition, enteric glia release immune regulators such as palmitoylethanolamide (PEA) in the presence of HIV-1 Tat, which exerts an anti-inflammatory effect through peroxisome proliferator–activated receptor alpha (PPARα) receptor signaling. PEA/PPARα signaling supports the intestinal immune response, reduces local inflammation, and lessens the functional consequences of the infection (170).

### 4.5. Enteric Glia in Necrotizing Enterocolitis

Necrotizing enterocolitis (NEC) is a severe condition that affects premature infants and is characterized by abdominal distention, bloody stools, and intestinal gas (171). The pathogenesis of NEC is poorly understood, but abnormal enteric glial numbers in both humans and animal models suggest roles for glia. Depleting enteric glia in mice increases NEC severity, and this effect is reversed by administering brain-derived neurotrophic factor (BDNF), a mediator secreted by glia that improves intestinal motility (109). Effects on glia in NEC appear to be mediated by TLR4 (172). Deleting glial TLR4 receptors in glia protects against glial loss and reduces disease severity in mice. These observations reinforce the idea that enteric glia contribute to the onset of NEC and could be a therapeutic target (82).

### 4.6. Enteric Glia and Gastrointestinal Cancer

Colorectal cancer is the third most commonly diagnosed cancer and represents 10% of cancer-related deaths worldwide (173). The onset of colorectal cancer is triggered by aberrant proliferation of the stem cells at the base of epithelial crypts. As described earlier, enteric glia play a role in maintaining homeostasis and renewal of the intestinal epithelium by influencing epithelial stem cell proliferation (33, 54, 111). Alterations in enteric glial function and changes in glial GFAP expression are correlated with tumor presence and severity in the gastrointestinal tract (174–176). The tumor microenvironment is densely populated by enteric glia, which display extensive arborizations that infiltrate tumors (177). Bidirectional communication between enteric glia and colonic cancer cells promotes a protumorigenic glial phenotype that enhances the tumorigenesis of cancer stem cells in vitro through glial PGE2 release and actions on EP4 receptors expressed by cancer cells (177). These mechanisms function to enhance tumorigenesis in models of colonic adenocarcinoma (178). Enteric glia also contribute to neuroendocrine tumor development in the intestinal tract through mechanisms involving the tumor suppressor protein menin (179). Deleting *Men1* in *Gfap*^+^ cells causes these cells to promote neuroendocrine tumors in the gut and pancreas, which suggests a glial or neural crest cell origin for these tumors. Interestingly, depleting *Gfap*^+^ cells also reduces tumor burden by 90% in mouse models of colonic cancer (176). Here, enteric glia seem to play a more prominent role during early stages of tumor development, while glial depletion at later stages does not affect tumor burden. Roles of enteric glia in the colorectal tumor microenvironment were covered in a recent review, and readers are referred there for additional information (137).

### 4.7. Enteric Glia and Extraintestinal Diseases

Enteric glia are also gaining interest for their potential contributions in broader neurodegenerative disorders such as PD (180). Gastrointestinal symptoms are common in PD and include hypersalivation, dyspepsia, constipation, abdominal pain, and defecatory dysfunction. Central glia play well-known roles in PD, and similar functions have been proposed for enteric glia in gut manifestations of the disease. Enteric glia display indications of a reactive phenotype such as increased GFAP expression in the 6-hydroxydopamine and 1-methyl-4-phenyl-1,2,3,6-tetrahydropyridine (MPTP) animal models of PD (181, 182). Glia also exhibit an accumulation of α-synuclein in these models (180). Interestingly, glial reactivity in the gut does not correlate with α-synuclein and occurs whether neurotoxic drugs are administered in the brain or periphery. These observations seem to indicate that glial reactivity is a reaction to inflammation rather than being a primary cause of disease. Data from human samples agree with this concept and show that enteric glia increase in proinflammatory markers such as IL-6, IL-1, TNF-α, GFAP, and S100B in PD (183, 184). Mucosal glia also exhibit abnormal cytoskeletal function due to changes in GFAP phosphorylation (183). These findings suggest that enteric glial reactions could contribute to the early stages of PD that are associated with intestinal inflammation, but more work is needed to understand exactly how (180).

## 5. SUMMARY

Enteric glia contribute to diverse processes directed at maintaining digestive homeostasis. They support and modulate neuronal functions, influence intestinal motility, preserve epithelial integrity, and regulate gut immune response. These roles are fulfilled through complex interactions with their surrounding cells and environment. Enteric glial biology remains an active area of research, and new data continue to expand the repertoire of functions that involve glia. Despite this, many questions remain regarding the basic cell biology of enteric glia, their pathways of intercellular communication with the other cells, and the roles glia play in common diseases such as IBD and IBS. Enhancing our understanding of these processes is a promising area for future research and may lead to innovative treatments for gastrointestinal disorders.

## Figures and Tables

**Table 1:** Glial and neuronal markers

Cellular subtype		Principal chemical markers or neurotransmitters		Additional chemical markers	Potential additional genetic markers	References
Enteric glia	Intraganglionic glia	GFAP, S100B, Sox10		Asc2, LPAR1, B-FABP, vimentin, PLP1/DM-20?	*Plp1*, *Slc18a*	12, 13, 15, 16,33, 40, 58
	Extraganglionic glia	S100B, Sox10				
Enteric neurons	Excitatory motoneurons	Hu C/D, peripherin, PGP9.5, tubulin βIII	Ach	GABA, calretinin, substance P, enkephalins, tachykinin	*Gfra2, Oprk1, Htr4, Piezo1*	185–188
	Inhibitory motoneurons		NO, VIP, ATP	Neuropeptide Y, GABA	*Ass1, Gfra1, Etv1*	185–188
	Ascending interneurons		Ach, ATP	Calretinin, substance P, enkephalin, tachykinin	Not determined	185–188
	Descending interneurons		Ach, NO, somatostatin, ATP	VIP, 5-HT, GABA	*Gad2*	185–187
	IPANs		Ach, CGRP	Calbindin, calretinin, substance P, advillin, neuromedin U	*Nmu, Nog, Dix3*	185–187
	Secretomotor/vasodilator neurons		VIP/Ach	Calbindin, neuropeptide Y, somatostatin, CGRP, calretinin	*Glp2r*	185–187
	IFANs		Ach	nNOS, calbindin, enkephalins, substance P, VIP, GRP, CKK	*Cck, Cartpt*	185–187, 189

Abbreviations: 5-HT, serotonin; Ach, acetylcholine; Asc2, astrocyte cell surface antigen 2; ATP, adenosine 5′-triphosphate; B-FABP, fatty acid–binding protein 7; CGRP, calcitonin gene–related peptide; CKK, cholecystokinin; GABA, gamma-aminobutyric acid; GFAP, glial fibrillary acidic protein; GRP, gastrin-releasing peptide; IFAN, intestinofugal neuron; IPAN, intrinsic primary afferent neuron; LPAR1, lysophosphatidic acid receptor 1; nNOS, neuronal nitric oxide synthase; NO, nitric oxide; PGP9.5, protein gene product 9.5; PLP1, proteolipid protein 1; S100B, S100 calcium-binding protein beta; Sox10, SRY-box transcription factor 10; VIP, vasoactive intestinal peptide.

**Table 2:** Glial phenotype, stimulation mechanisms, and consequences in pathophysiology

Condition		Glial phenotype	Known mechanism(s) of glial stimulation	Downstream consequences	Reference(s)
Homeostasis		Active	Cholinergic, purinergic, cytokine signaling, mechanical stimuli	Neuronal support and maintenance Modulation of gastrointestinal motility Maintenance and regulation of intestinal epithelium function Control of immune homeostasis within the intestine	8–12, 31, 33, 35–39, 53–55, 60, 67–70, 74–77, 79–83, 93, 96, 99–122, 127–129, 131–136
Inflammation	Irritable bowel syndrome/inflammatory bowel disease	Reactive	Proinflammatory environment	↑ GFAP expression and secretion of IL-1β, IL-6, IL-10, IFN-γ, TNF-α Alteration of GDNF, PGE2, GSNO, 15dPGJ2, 15HETE, and ATP secretion ↑ NO production and ATP release through connexin-43 Abnormal neuronal activation and neuronal death	55, 87, 109, 111, 114, 140–143, 145–149
	Visceral pain	Reactive	Proinflammatory environment	↑ IL-1β and IL-6 secretion Influence macrophage recruitment and phenotype through M-CSF signaling Sensitize nociceptive nerves through PGE2/EP4 signaling	127, 149, 153
Dysmotility disorder	Postoperative ileus	Reactive	IL-1/IL-1-R1 signaling, sympathetic β-adrenergic signaling, endothelin-1/endothelin B signaling	Alteration of glial gene expression in two phases: (*a*) proinflammatory and (*b*) proproliferation and migration ↓ intestinal contraction and peristalsis through endothelin B signaling	144, 154, 155, 157
	Chronic intestinal pseudo-obstruction	Reactive	LPA/LPAR1 signaling decrease	Changes in ENS architecture Abnormal gastrointestinal motility	159
Infection	*Heligmosomoides polygyrus*	Reactive	IFN-γ/IFNr2 signaling	Glial secretion of CXCL10 ↑ tissue repair	118
	Chagas disease (*Trypanosoma cruzi*)	Reactive and gliosis	Unknown	↓ glial cells in the dilated portion ↑ GFAP expression in the nondilated portion ↑ glial antigen-presenting molecules	162, 163
	*Clostridium difficile*	Reactive and gliosis	*Clostridium difficile* toxins	↑ glial senescence and death ↑ S100B/RAGE signaling ↑ S100B and *IL-6* expression ↓ neutrophil recruitment	164–166
	Human immunodeficiency virus	Reactive	HIV1-Tat, TLR4 signaling	↑ GFAP, S100B, and iNOS expression ↑ PEA/PPARα signaling to support immune response and reduce inflammation	169–170
Necrotizing enterocolitis		Reactive and gliosis	TLR4 signaling	Glial loss ↓ intestinal BDNF concentration ↑ disease severity and intestinal dysmotility	172
Cancer	Colon cancer	Reactive	Bidirectional communication with cancer cells	Protumorigenic glial phenotype ↑ tumorigenesis in colonic adenocarcinoma through PGE2/EP4 signaling	177, 178
	Neuroendocrine cancer		Gastrin/cholecystokinin B receptor signaling	↑ gastrin secretion and menin-1 degradation ↑ intestinal neuroendocrine tumor development	179, 190
Parkinson’s disease		Reactive	TLR2/4 signaling, proinflammatory environment	↑ IL-6, IL-1β, TNF-α, GFAP, S100B ↓ GFAP phosphorylation Synaptic dysfunction and changes in colonic excitatory motility	183, 184

Abbreviations: 15-HETE, 15-hydroxyeicosatetraenoic acid; 15dPGJ2, 15-deoxy-Δ-^12,14^-prostaglandin J2; ATP, adenosine 5′-triphosphate; BDNF, brain-derived neurotrophic factor; CXCL10, chemokine interferon-γ inducible protein 10; ENS, enteric nervous system; GDNF, glial cell line–derived neurotrophic factor; GFAP, glial fibrillary acidic protein; GSNO, *S*-nitrosoglutathione; HIV1-Tat, human immunodeficiency virus-1 transactivating factor; IFN-γ, interferon gamma; IFNr2, interferon receptor 2; IL, interleukin; IL1-R1, interleukin-1 receptor 1; iNOS, inducible nitric oxide synthase; LPA, lysophosphatidic acid; LPAR1, lysophosphatidic acid receptor 1; M-CSF, macrophage colony–stimulating factor; NO, nitric oxide; PEA, palmitoylethanolamide; PGE2, prostaglandin E2; PPARα, peroxisome proliferator–activated receptor alpha; S100B, S100 calcium-binding protein beta; TLR, Toll-like receptor; TNF-α, tumor necrosis factor-alpha.

## SUMMARY POINTS

1. Enteric glia play crucial roles in maintaining digestive homeostasis and functions.
2. Enteric glia are heterogeneous and display phenotypic plasticity throughout the gut.
3. Bidirectional communication between neurons and glia supports and enhances neuronal functions.
4. Enteric glia modulate gut immune responses with impacts on the inflammatory environment.
5. Environmental cues alter the phenotype of enteric glia, and reactive glial responses are involved in gut disease processes.
6. Enteric glial mechanisms and signaling pathways are involved in digestive tract disorders, underlying their importance in gastrointestinal health. Enteric glia could be a promising therapeutic target for treating gastrointestinal disorders.
